# Future research focus and trends in nerve transplantation: a bibliometrics research from 2015 to 2024

**DOI:** 10.3389/fneur.2025.1581859

**Published:** 2025-05-22

**Authors:** Sheng Wang, Jiajia Lu, Hui Han, Yongchuan Li, Nan Lu, Aimin Chen

**Affiliations:** Department of Traumatic Orthopedics, Shanghai Fourth People's Hospital, School of Medicine, Tongji University, Shanghai, China

**Keywords:** nerve transplantation, lumbosacral nerve, lower limbs, sacral nerve, bibliometrics

## Abstract

**Background:**

Nerve transplantation, as a classical nerve repair technique, has received extensive attention in recent years. However, the rapid development of this field has also brought challenges such as knowledge fragmentation and the blurring of research hotspots. Therefore, at present, the future research direction of this field is not clear. Through the research method of bibliometrics, this study investigates the research hotspots and development trends in this field, and fills the research gap in this field.

**Methods:**

The publications in the core database of Web of Science (WoS) were collected, and the included publications were analyzed by bibliometric methods after the screening exclusion process. The basic information of the publications was analyzed, and the future development direction and research hotspots were predicted and visualized.

**Results:**

A total of 847 publications were included in the research, in the past 10 years, the number of publications in this field has nearly doubled (49 publications), and the number of publications in this field will continue to grow in the next 15 years. In terms of national contribution, the United States is the largest contributor (289 publications), and in terms of institutional contribution, Fudan University is the largest contributor (47 publications).

**Conclusion:**

This is the first bibliometric analysis in the field of nerve transplantation, which included all publications in this field in the past decade. Our results illuminate the contributions of countries and institutions, international cooperation, interdisciplinary relationships, and future research directions. This research will point out the future development path of nerve transplantation.

## 1 Introduction

Nerve transplantation, as a classic nerve repair technique, has attracted widespread attention in the field of regenerative medicine in recent years. It transplants healthy nerves into the damaged nerve sites, promoting the regeneration of damaged nerves and the recovery of their functions, offering hope for the treatment of nerve injuries (1, 2). Compared with nerve decompression and in-situ nerve anastomosis, the application of nerve transplantation can solve more complex clinical problems, such as nerve transection caused by severe sacral fractures cannot be solved by simple decompression surgery, and because the severed nerve will retract, it is difficult to find the broken end and anastomosed, which also limits the application of *in situ* nerve anastomosis, and this is also widely used in various clinical disciplines (neurosurgery, traumatic orthopedics, microsurgery) (3). Firstly, in terms of motor and sensory dysfunction of the upper and lower limbs, nerve transplantation surgery has always been an effective method. For example, in the treatment of brachial plexus avulsion injury, the use of the healthy side of the neck nerve as a donor transplantation has been proved to be effective in clinical verification (4). And for such as facial paralysis, headache, and dry eye caused by nerve causes, nerve transplantation surgery has a certain effect, previous studies have confirmed that facial nerve transplantation can treat facial paralysis caused by large-area defects of the facial nerve (5). More importantly, lower limb nerve injury often leads to urinary disorders, defecation disorders and sexual dysfunction, and this technique also plays an advantage in treating such injuries. Using the sural nerve or healthy lumbosacral nerve as a donor can effectively solve the above problems (6, 7). However, precisely because nerve transplantation is widely used and involves multiple disciplines, which makes the research results in this field numerous and scattered, it is difficult for researchers to have a comprehensive understanding of the current status and trends in the field, which also limits the communication between different disciplines to a certain extent. Therefore, it is crucial to have a quick and comprehensive understanding of the current status and trends of nerve transplantation.

Bibliometrics, as a scientific evaluation tool based on quantitative analysis, can systematically sort out the knowledge structure in the field of nerve, identify research trends, evaluate the academic influence of countries and institutions, and provide data-driven decision support for future research directions (8). Therefore, this research method can help researchers quickly understand the overall development context of a field, identify key research results and research directions, and thus provide valuable information for research in this field, which is highly scientific (9).

The ultimate goal of nerve transplantation is to achieve precise repair and reconstruction of nerve function, and the realization of this goal cannot be separated from the grasp of (the developmental laws of the field). Through the scientific big data analysis of bibliometrics, we are able to transcend the limitations of individual studies and achieve overall control of this research field (10). The purpose of this research is to use bibliometric methods to comprehensively analyze the publications in the field of nerve transplantation, reveal the research hotspots, development trends and potential research gaps in this field. This not only helps to provide guidance for the research direction of researchers, but also provides a scientific basis for clinical application and policy formulation.

## 2 Materials and methods

### 2.1 Data screening and inclusion

**Figure 1:**
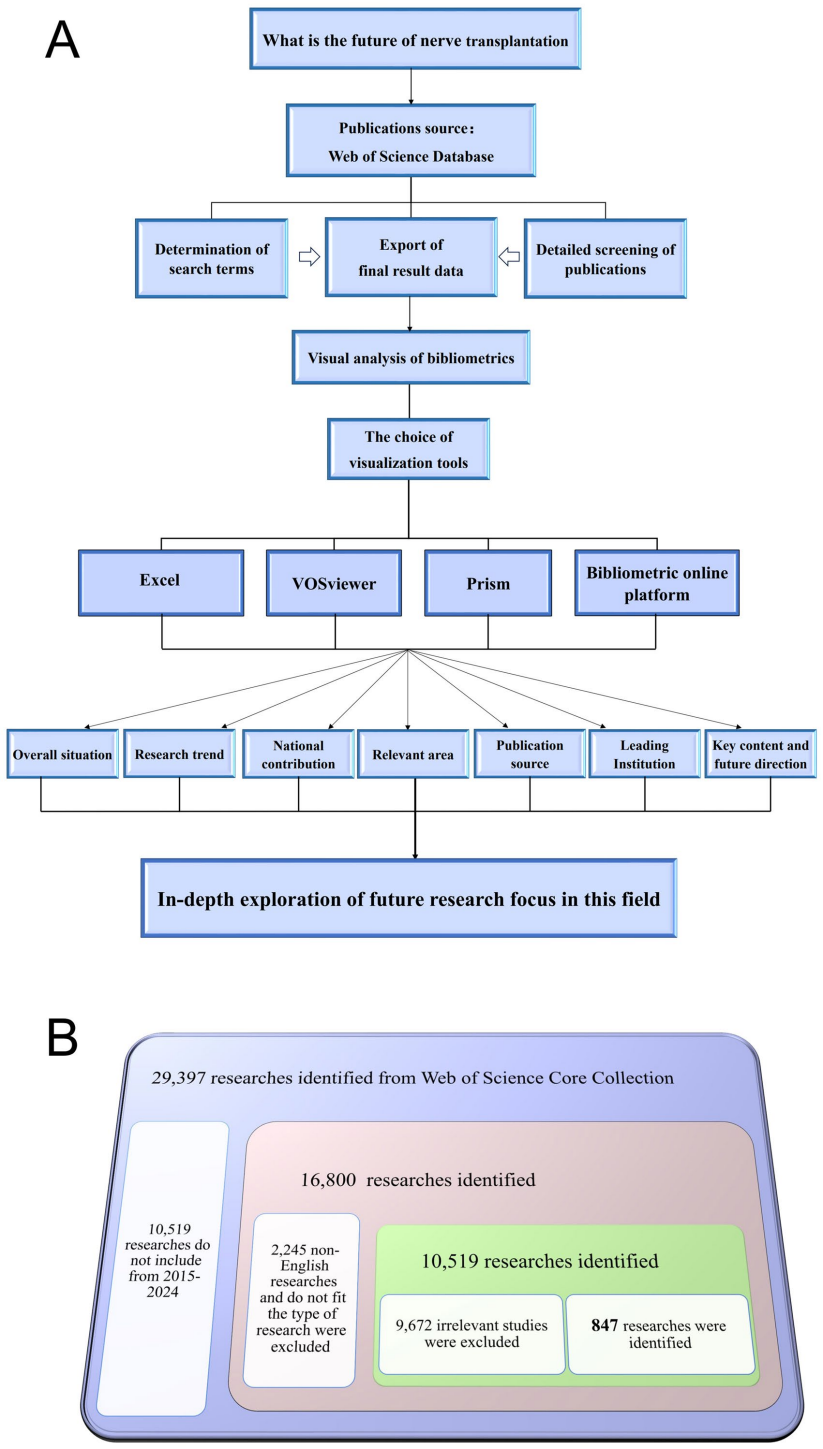
Flow diagram of the research process. **(A)** Research process. **(B)** The specific process of screening publications.

We took the core database of Web of Science as the core content of our search, and nerve transplantation as the research theme. In order to avoid an incomplete search, the search formula was set as (((TS = (Nerve transfer)) OR TS = (Nerve graft)) OR TS = (Nerve transposition)) OR TS = (Reinnervation), and the search time was January 1, 2025. Through the search, we found a total of 29,397 publications related to nerve transplantation, and then we conducted intelligent and manual searches, and the time of the publications was the last 10 years (2015–2024). After excluding unrelated research, a total of 12,764 publications met the requirements. After excluding non-English publications and publications that did not match the article type, a total of 10,519 publications were included in the research. Finally, after manual screening, 9,672 unrelated publications (publications that mentioned the term neural transplantation in the research but were not focused on neural transplantation) were excluded, and a total of 847 publications were included in the research. The above process was conducted by two authors, and any disagreements were resolved by the experienced corresponding author. The entire inclusion process is shown in Figure 1A.

### 2.2 Bibliometrics analysis

The use of scientific methods to analyze the research directions and potential hotspots in a specific field of study is a feature of bibliometrics. In this research, we adopted visualization tools for analysis, in which Prism and Excel were used to describe the future research trends and publication trends in this field while VOSviewer was used to deeply analyze the contributions of countries, journals, institutions and the keywords, at the same time, we used an online bibliometric analysis platform to evaluate international cooperation relationships (11, 12). Finally, we comprehensively presented the research situation in the field of nerve transplantation in the past 10 years, focusing on the aspects of overall publishing, national contributions and cooperation networks, contributions of publishing institutions, sources of publications, keyword clustering, and identification of future research hotspots.

### 2.3 Mining and summary of future research hotspots

Based on the bibliometrics analysis of the field and research trends in neural transplantation, we conducted a comprehensive analysis of relevant publications to explore the current research and emerging research areas in neural transplantation. We summarized the existing research results and made reasonable predictions for the future, laying a foundation for further research in this field. The research process is shown in Figure 1B.

## 3 Results

### 3.1 Overall publication trend (research popularity)

**Figure 2:**
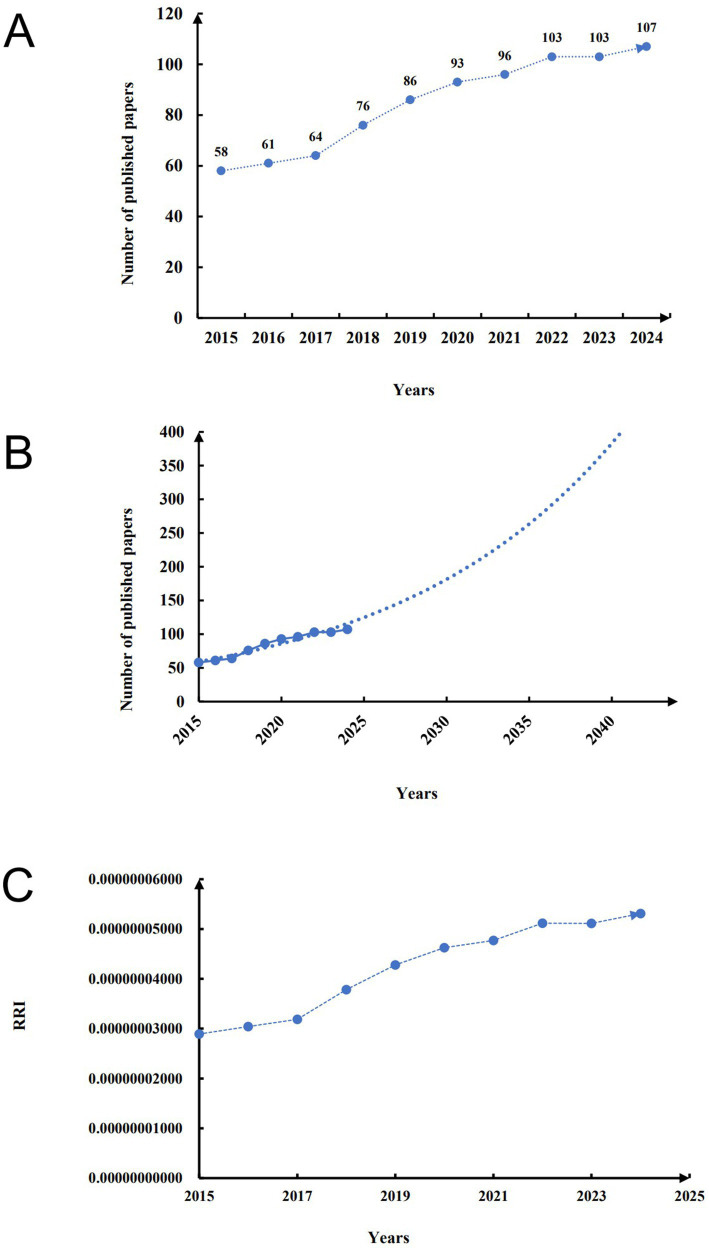
Flow diagram of overall publication trend. **(A)** The number of publications in this field in the last 10 years. **(B)** Research trends in this field in the next 15 years. **(C)** RRI in the last 10 years.

When studying the development status and research popularity of a field, the number of publications serves as the most important index. From 2015 to 2024, the number of publications in the field of neural transplantation has been increasing year by year, with the highest number of publications in 2024, totaling 107 articles. In the past decade, the number of publications in this field has increased nearly 1 time (Figure 2A). Based on the current trend of publications, we used a fitted curve to predict the number of future publications in this field, and the results suggested that the publications in this field will continue to increase in the next 15 years (Figure 2B). Relative research interest (RRI) represents the relative popularity of research in a field, calculated as the total number of publications in a field/the total number of articles included in the Web of Science core database in the same year. We find that the RRI value in the field of neural transplantation has a slight increase (Figure 2C), which represents an increase in the degree of attention in this field.

### 3.2 National contributions and partnerships

**Figure 3:**
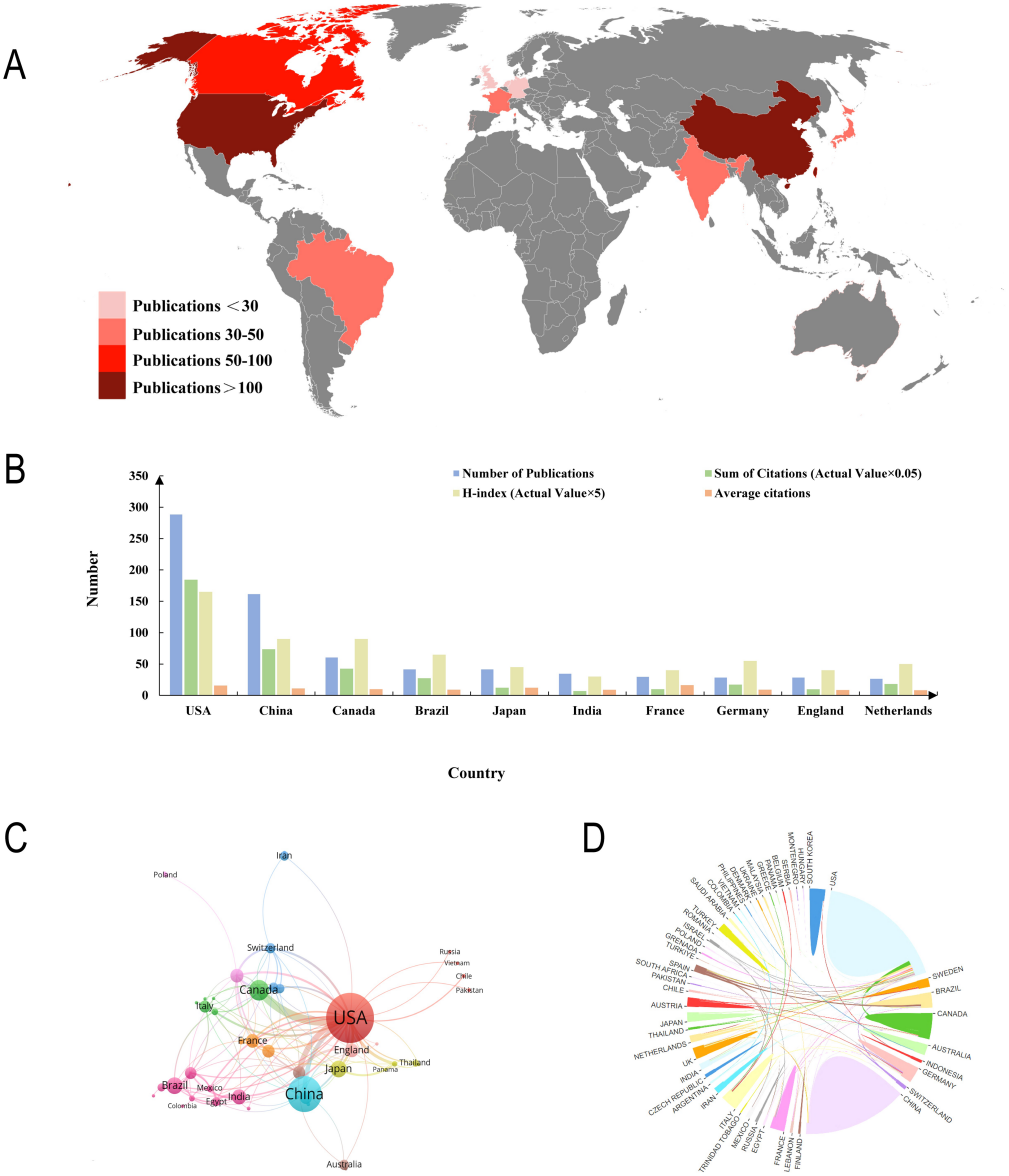
Contributions of different countries to the research. **(A)** Overall situation of number of publications worldwide. **(B)** Number of publications, citation frequency (×0.05), H-index (×5), and average citations in the top 10 countries or regions. **(C,D)** The number of national publications and the cooperative relations between them.

We conducted a visual analysis of the number of publications by each country. In terms of the number of publications, the United States is the largest, followed by China. Although other countries also contributed, the number of their publications was relatively low (Figures 3A,B). Specifically, the United States, as the country with the most published papers, published 289 publications, ranking first in both the H-index (33) and the average number of citations (12.62). China ranked second in the number of publications (162) and was slightly lower than the United States in both the H-index and the average number of citations. Other countries with significant contributions include: Canada, Brazil, and Japan. At the same time, the huge contribution of the United States in this field is also reflected in the cooperation between countries, which further emphasizes the leadership of the United States (Figures 3C,D). More in-depth research needs to be carried out in this field under the leadership of the United States in the future.

### 3.3 Contribution of the issuing institution and journal contribution

**Figure 4:**
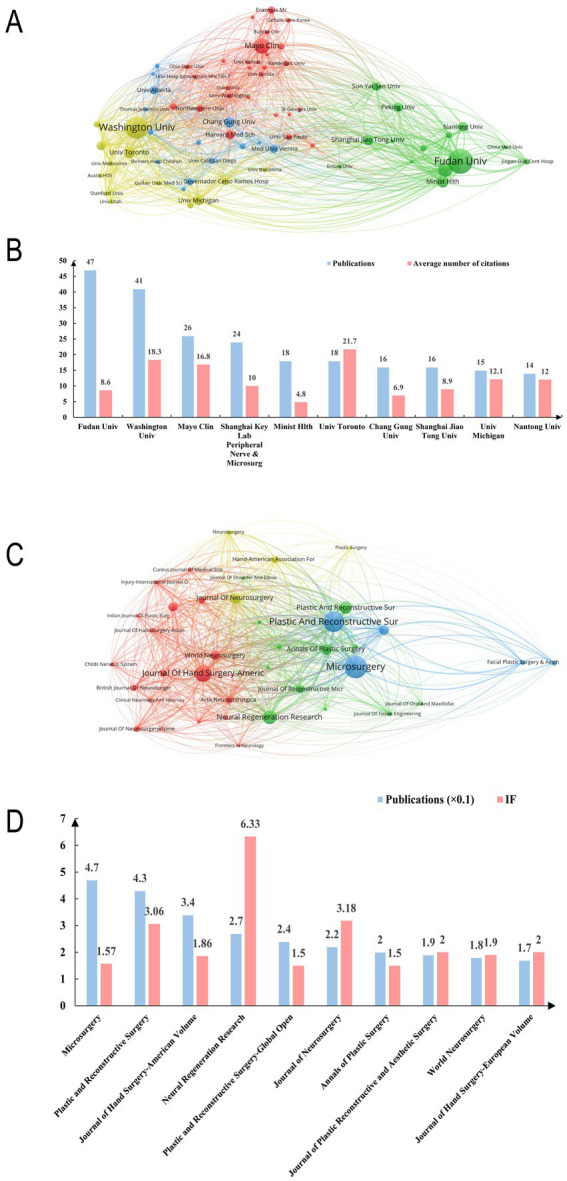
Contribution of institutions and journals in this field. **(A)** Network of institutions visualized in VOSviewer. The size of circles represents the number of publications. **(B)** The number of publications and average citations of the top 10 institutions in the field. **(C)** Network of journals visualized in VOSviewer. The size of circles represents the number of publications. **(D)** The number of publications and impact factors of the top 10 journals in the field.

In bibliometrics, institutions with high contributions are often affiliated with high-contributing countries. And the contribution of institutions to the field is also an important part of bibliometrics. We intuitively represent the contribution of publishing institutions by visualization (Figure 4A). Among the top 3 institutions, 2 were located in the United States and 1 in China. Notably, although Fudan University ranked first in the number of publications (47), its average citation rate (8.6) was significantly lower than that of institutions in the United States, suggesting that the research quality and influence of publications in the United States were stronger (Figure 4B).

The journal contribution refers to the number of publications included in our study that are indexed by a specific journal. The choice of journals often reflects the cooperative relationship between fields. We analyzed the 10 highest-volume journals in the field of nerve transplantation and their impact factors in 2024 (Figures 4C,D). Among these 10 journals, we found that 3 journals were related to neurosurgery, 3 journals were related to microsurgery and hand surgery, and 4 were related to reconstructive surgery, which represents that the field of nerve transplantation involves multiple disciplines and requires multidisciplinary cooperation to achieve.

### 3.4 Keywords clustering and future research hotspots

**Figure 5:**
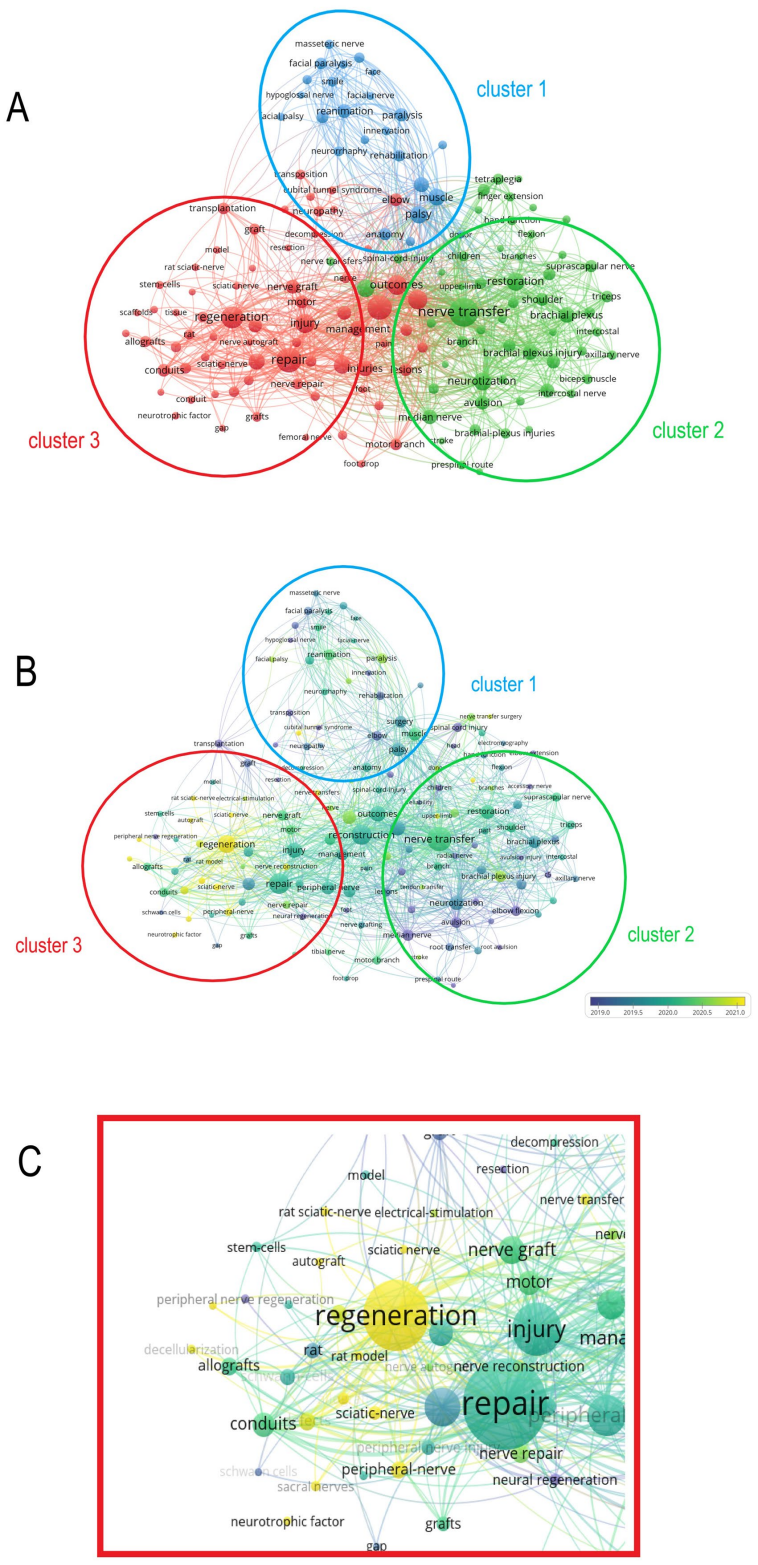
Analysis of keywords in all publications. **(A)** Mapping of the keywords in the field of nerve grafting. The size of the circle represents the frequency of the keyword, and different colors represent different clusters. **(B)** Distribution of keywords according to the average time of appearance. Blue represents an early appearance, and yellow represents a late appearance. **(C)** The latest cluster of keywords, the latest keyword is sacral nerve.

Keyword analysis is the most important point in bibliometrics which often summarizes the current research focus of a field and can predict future research hotspots. After a macro-level analysis of the key relevant fields it becomes crucial to identify which keywords will become the focus of future research. We classified 148 keywords that appeared more than 10 times in 847 publications (Supplementary Table 1). The research areas involved are divided into three groups each representing a different research area of neural transplantation. As shown in Figure 5A: the blue circle (cluster 1) is related to cranial nerves and the keywords include facial nerve (9 times) masseteric nerve (17 times) facial paralysis (28 times) smile (20 times) and hypoglossal nerve (11 times). Green circle (cluster 2) involves keywords related to the upper limb nerves including axillary nerve (17 times) biceps muscle (1 times) C7 transfer (10 times) intercostal nerve (16 times) and median nerve (51 times). Red circle (cluster 3) is related to the lower limb nerves including the keywords: sciatic nerve (19 times) sacral nerves (14 times) foot drop (11 times) tibial nerve (18 times) femoral nerve (10 times)

In addition to the cluster analysis of keywords, we also evaluated the most concerned keywords in recent years. As shown in Figures 5B,C, the lighter colored areas are mainly concentrated in cluster 3. This indicates that the keywords in cluster 3 are relatively new, and also foreshadows that the related research of lumbosacral nerves will become a research hotspot in this field in the future.

## 4 Discussion

### 4.1 Research trends of neural transplantation

The number of publications in the field of nerve transplantation is expected to continue to increase over the next 15 years, indicating that scholars in various countries are increasingly interested in this field of research, and the steady increase in the RRI also shows that nerve transplantation has received more attention in recent years compared to other. In terms of contribution, the United States leads the way in the number of publications. Similarly, in terms of research quality, the United States stands out, which may be attributed to the contributions of renowned institutions such as the University of Washington and the Mayo Clinic. The reason for the huge contribution of the United States may be that the cooperation between countries is relatively close, and the United States has the most international cooperation, and interdisciplinary cooperation is essential. Meanwhile, the earlier start, larger scientific research investment and obvious clinical transformation advantages of the United States further consolidate its leadership position in this field. After analyzing the journals, we found that the journals in the field of nerve transplantation include topics such as neurosurgery, microsurgery, hand surgery and reconstructive surgery, which also represents that the development of this field requires the multidisciplinary collaboration to be realized. Neurosurgery was the first discipline to develop end-to-end nerve anastomosis. The perineurium suture technique was explored to reduce the misdirection of axons during nerve regeneration, which laid the foundation for the “pathway reconstruction” of nerve transplantation (11). Microsurgery can realize the delicate operation of nerve transplantation with the help of operating microscope. Hand surgery has established the technique of “multiple nerve transfer,” such as transferring accessory nerve and intercostal nerve to the site of brachial plexus nerve injury to restore shoulder abduction, elbow flexion and other functions, which promotes the clinical practice of peripheral nerve transfer (12, 13). In reconstructive surgery, collagen, polylactic acid-glycolic acid and other materials are used to construct nerve conduits, filled with Schwann cells and nerve growth factors, etc., to simulate the nerve microenvironment, replace traditional autologous nerve transplantation, solve the problems of limited donor and donor site complications, and expand the application range of nerve transplantation (14). These disciplines play their unique advantages in the field of nerve transplantation, and jointly promote the progress of this field through technical intersection, theoretical complementarity and clinical collaboration.

### 4.2 Research clusters of nerve transplantation

In the research of bibliometrics, the most important part is the analysis of keywords, and we found that after the analysis of keywords, the current research in this field can be roughly divided into three major clusters, namely: cranial nerves, upper limb nerves and lower limb nerves. After a detailed analysis, it was found that the research in the field of lower limb nerves has become the focus and hotspots of research in recent years.

In terms of cranial nerve transplantation, many functions can be restored through cranial nerve transplantation, for example, the transplantation of the hypoglossal nerve, facial nerve and the motor branch of the trigeminal nerve can effectively treat facial paralysis (15–17). And recent studies have found that this surgery can also enable patients to smile again (16). Researches have also shown that abducent nerve grafting can treat abducent paralysis of the eye and achieve the re-movement of the eye. At the same time, the study found that the facial nerve can control tear volume, and nerve transplantation can effectively treat dry eyes. Therefore, the transplantation of cranial nerve is able to treat the clinical symptoms of patients and has a clear effect (18).

Another area of research covered in the research is the upper limb nerves. Back in 2018, a research on C7 nerve transplantation to treat spastic paralysis of the upper limbs was published in the journal New England, causing a stir in the transplant medicine field (14). In addition to the transplantation of cervical nerves (19), we found that the median nerve, radial nerve, and ulnar nerve can all be transplanted to treat motor and sensory disorders of the upper limb, such as claw hand, radial nerve rupture, and median nerve damage, all of which can be treated with nerve transplantation to restore function (20, 21).

**Figure 6:**
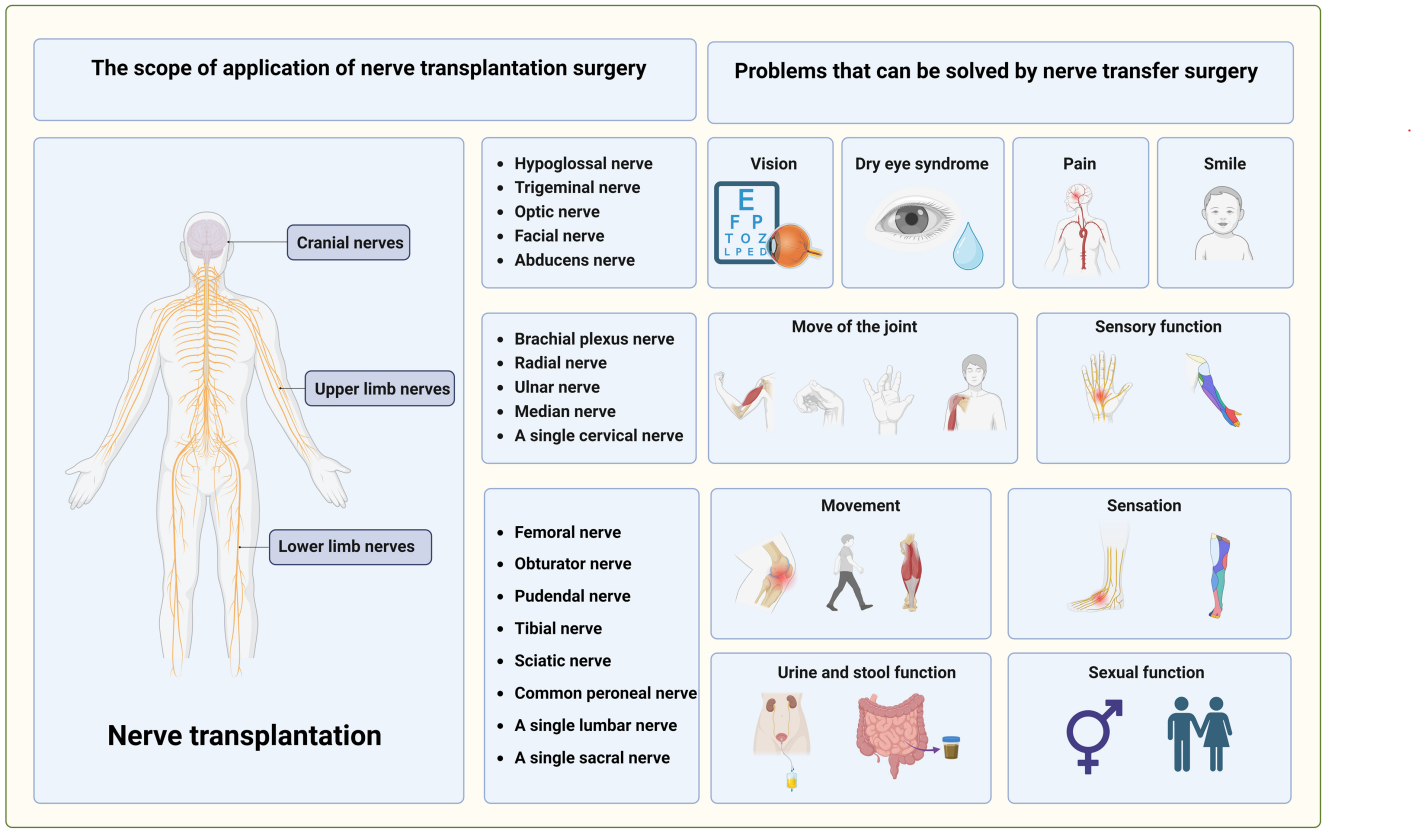
The main application range of nerve transplantation and the clinical problems that can be treated.

Similarly, lower limb nerves have attracted more attention in recent years. In addition to the transplantation of a single lumbar and sacral, its branches, such as the tibial nerve, common peroneal nerve, femoral nerve, and obturator nerve, can also be transplanted. Compared with the upper limb nerves, lumbosacral nerves control the human body’s urination, defecation, and sexual functions in addition to motor and sensory functions, and nerve transplantation can regulate these functions, thereby improving the quality of life (22, 23) (Figure 6).

At present, the bridging materials for nerve transplantation mainly include nerve autografts, allografts and nerve conduits. For small nerve defects, tension-free suture is feasible, but for large nerve defects, autologous nerve transplantation is still the first choice of clinical treatment. For long or large nerve defects, due to the limited source of nerve grafts, autologous nerve transplantation cannot meet the clinical needs, and it is prone to complications such as neuroma formation in the donor site, motor and sensory dysfunction, and eventually cause new nerve injury. Therefore, allografts or catheters are widely used in clinical practice (24). It has been shown that the overall rate of meaningful recovery of sensory and motor function in autografts and allografts did not differ significantly between short-and long-gap nerve injuries. However, the meaningful recovery rates for autograft and allograft repairs were significantly higher compared with conduits in sensory short gap repairs. Complication rates were comparable for autograft and allograft but higher for conduit with regard to pain (25, 26). With the progress of tissue engineering technology, the development of nerve conduits with high biocompatibility, low immunogenicity, satisfactory degradability, appropriate aperture size, good mechanical properties, and high efficiency to promote regeneration of injured nerve is the focus of future research in the field of nerve transplantation (14).

### 4.3 Sacral nerve: future research direction of nerve transplantation

We sort the keywords according to the order of their appearance, with the latest keywords in light yellow, and it is found that the sacral nerve is the latest keyword, which indicates that the focus of nerve transplantation research is beginning to focus on the lower limbs, and the sacral nerve will be the focus of future research. It is worth noting that the sacral nerve, as one of the nerves that innervate the lower limb movement and sensation, also has the function of controlling urination defecation, and sexual function. These functions are not found in the cervical nerve, thoracic nerve, lumbar nerve and their branches, which can also indicate that the emphasis on this field in recent years is meaningful.

**Figure 7:**
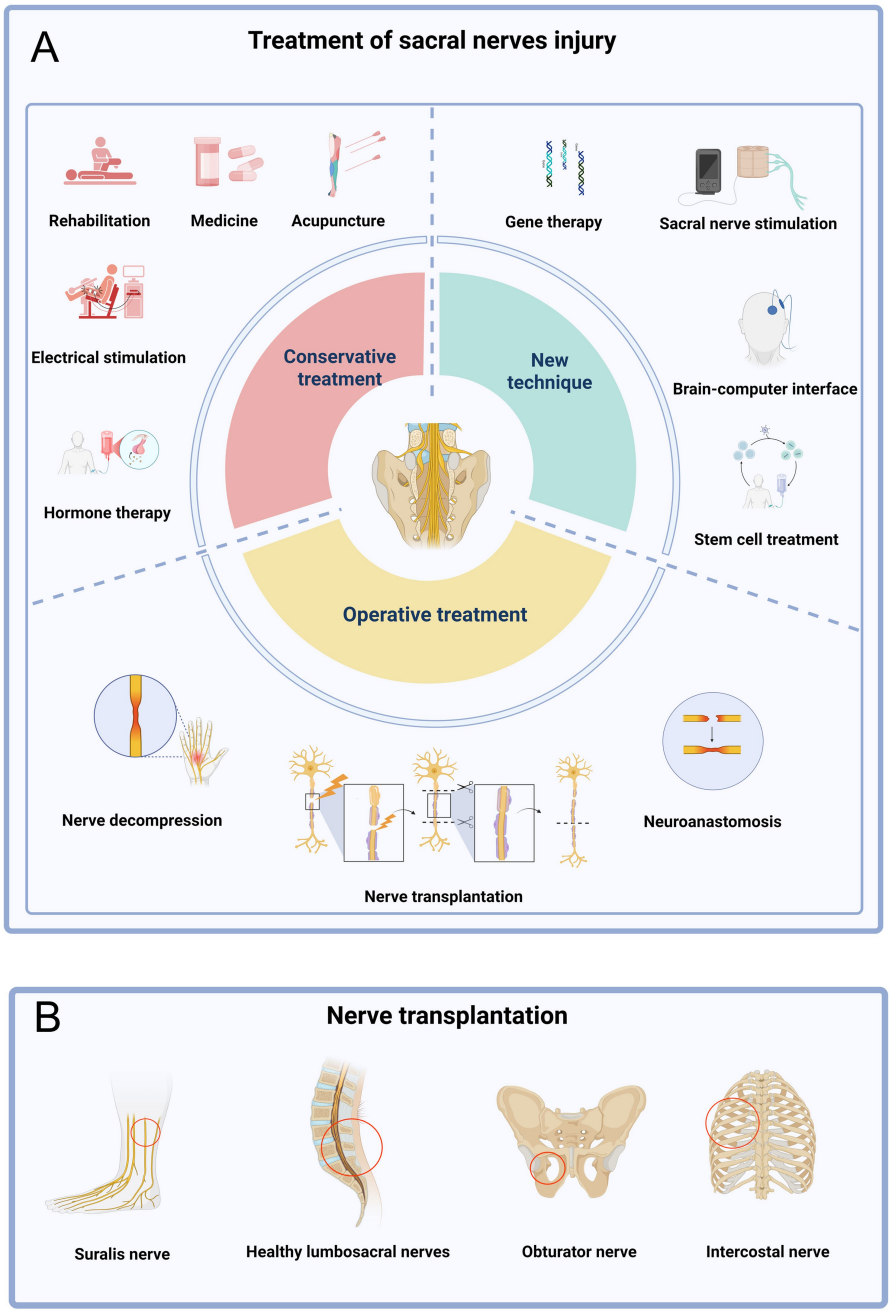
Treatment of sacral nerve injury. **(A)** The treatment of sacral nerve injury is mainly divided into conservative treatment, operative treatment and new technology. **(B)** Donor nerve for sacral nerve transplantation.

For sacral nerve injury, a variety of treatment methods have emerged in recent years, and some non-surgical treatments, such as hormonal stimulation, electrical stimulation, nerve growth factor, and physical therapy, have played an important role in the treatment of some sacral nerve injuries (27, 28). In recent years, with the development of science and technology, gene therapy and stem cell therapy have gradually entered the field of vision of researchers, and at the same, with the development of medical engineering combination, some intelligent materials have also been applied in the field of sacral nerve injury treatment (29, 30). However, it is important to note that these conservative treatments sometimes cannot completely solve the problem of sacral nerve injury, especially when there is organic damage to the sacral nerve itself (such as rupture, compression, or complete degeneration), at which time surgical treatment becomes particularly important. The methods of surgical treatment include: nerve anastomosis, nerve decompression, and nerve transplantation (31) (Figure 7A).

However, nerve anastomosis is only suitable for tension-free docking of nerve stumps (the distance of the defect is usually < 5 mm). If the defect is too long (e.g., > 10 mm), forced anastomosis will lead to excessive tension of nerve stumps, affect blood supply and regeneration, and even cause scar hyperplasia at the anastomosis (32, 33). The use of nerve decompression surgery has a good effect on the treatment of compressed nerves, but not on the treatment of nerve rupture. Research in recent years has confirmed the application scope of sacral nerve transplantation is relatively wide. It breaks the limit of natural repair and simple anastomosis through structural intervention, and provides the possibility of active repair for long segment defects, complex fractures or avulsions. It is especially of irreplaceable value in preserving or reconstructing key functions such as bowel and bladder control and sexual function (34). For example, the contralateral nerve, the obturator nerve, the intercostal nerve, and even the sural nerve are all alternative choices for the sacral nerve. Repair of sacral plexus injury on the affected side using a healthy S1 nerve proved to be effective. This surgical approach not only preserves the function of the healthy side but also promotes the functional recovery of the affected side (35).

The researchers also used the less functionally disrupted? sural nerve for transplantation surgery, which can treat the patient’s transected sacral nerve thus solving the problem of radiating pain in the lower limb (36). Clinical cases also confirmed that the use of obturator nerve as a donor nerve for transplantation surgery, and the urination function of the patients recovered after the operation (37) It can be seen that the sacral nerve transplantation surgery is now widely used and will remain a hot topic for future research (Figure 7B).

However, there are still some challenges and issues in the treatment of sacral nerve injury by nerve transplantation. Firstly, this type of surgery has a high level of difficulty, significant trauma, and a longer recovery period for patients. In addition, long-term denervation also leads to atrophy of effectors such as bladder detrusor muscle, decreased receptor sensitivity, and limited functional recovery even if nerve transfer is successful (38). Some emerging rehabilitation techniques can be used as a powerful supplement to traditional surgery. It is worth mentioning that with the development and progress of technology, sacral nerve stimulation (SNS) has gradually been applied in clinical practice. This is a physical therapy method that acts on specific sacral nerves through stimulation current. It can be analogized to a heart pacemaker, which can control urination defecation (39). And brain-computer interface is also an important technological innovation in recent years, allowing the establishment of a control relationship between the human brain and the computer, which can replace the function of damaged nerves to a certain extent. The existing brain-computer interface technology can help paraplegic patients to stand, walk and even climb, which is extremely important for patients with lumbosacral plexus injury. These two techniques are extremely important for patients who have poor or inoperable surgery (40, 41) (Figure 7A).

## 5 Limitations

There are potential limitations to the study. First of all, regarding the selection of databases, it is more comprehensive to select multiple databases, such as Embase and PubMed, but the bibliometrics technology has requirements on the format of data, and the information of the above databases cannot be analyzed. Second, the exclusion of non-English publications may have had an impact on the overall results, but due to the small number of non-English publications, the impact on this accuracy is smaller. Finally, the bibliometrics summary of research hotspots in this field is completely credible, but there may be some errors in the prediction of future research.

## 6 Conclusion

Over the next 15 years, research interest in the field of nerve transplantation will continue to increase, and the United States, as the leader in the field, has a clear advantage in terms of the number of publications and international cooperation. Neurosurgery, microsurgery, hand surgery, and reconstructive surgery all played an important role in this study. At present, there are three research directions in this field, namely, cranial nerves, upper limb nerves and lower limb nerves. In recent years, the focus of research has shifted to the lower limb nerves. In particular, sacral nerve transplantation is becoming the latest core keyword and will be the core of future research in this field.

## Data Availability

The original contributions presented in the study are included in the article/Supplementary material, further inquiries can be directed to the corresponding authors.
