# Supplementary material for: Future research focus and trends in nerve transplantation: a bibliometrics research from 2015 to 2024
Source: Front Neurol. 2025 May 22;16:1581859. doi: 10.3389/fneur.2025.1581859 (PMC12137082; doi:10.3389/fneur.2025.1581859)
Supplement: Supplementary file 1 [file Table_1.docx]

**148 keywords that appear more than 10 times**

| **label** | **weight<Occurrences>** | **score<Avg. pub. year>** |
| --- | --- | --- |
| **allograft** | **16** | **2021.8125** |
| **allografts** | **25** | **2020.16** |
| **autograft** | **10** | **2021.5** |
| **axonal regeneration** | **24** | **2020.8333** |
| **conduits** | **37** | **2020.2432** |
| **decellularization** | **12** | **2020.9167** |
| **defects** | **25** | **2020.88** |
| **electrical-stimulation** | **10** | **2020.7** |
| **expression** | **11** | **2019.9091** |
| **functional recovery** | **39** | **2019.8462** |
| **gap** | **10** | **2019.3** |
| **graft** | **25** | **2019.2** |
| **grafts** | **20** | **2020.2** |
| **injury** | **91** | **2019.956** |
| **model** | **12** | **2019.8333** |
| **motor** | **36** | **2020.3056** |
| **nerve autograft** | **10** | **2020.5** |
| **nerve graft** | **44** | **2020.2273** |
| **nerve grafting** | **19** | **2019.5789** |
| **nerve injury** | **27** | **2019.8889** |
| **nerve reconstruction** | **17** | **2021.1176** |
| **nerve regeneration** | **63** | **2019.5238** |
| **nerve repair** | **27** | **2020.5185** |
| **neural regeneration** | **17** | **2017.5882** |
| **neurotrophic factor** | **11** | **2021.5455** |
| **peripheral nerve** | **67** | **2019.9104** |
| **peripheral nerve injury** | **20** | **2019.8** |
| **peripheral nerve regeneration** | **11** | **2018.1818** |
| **peripheral-nerve** | **24** | **2021.875** |
| **rat** | **21** | **2019.4762** |
| **rat model** | **12** | **2021.8333** |
| **rat sciatic-nerve** | **11** | **2021.1818** |
| **reconstruction** | **162** | **2019.8765** |
| **regeneration** | **128** | **2021.7266** |
| **reinnervation** | **47** | **2020.1702** |
| **repair** | **171** | **2019.9766** |
| **sacral nerves** | **14** | **2023.5143** |
| **scaffolds** | **10** | **2021.5** |
| **schwann cells** | **10** | **2019.2** |
| **schwann-cells** | **23** | **2019.8696** |
| **sciatic nerve** | **12** | **2022.9333** |
| **sciatic-nerve** | **19** | **2022.8789** |
| **stem-cells** | **14** | **2020** |
| **tissue** | **11** | **2019.9091** |
| **transplantation** | **28** | **2018.9286** |
| **accessory nerve** | **13** | **2018.3077** |
| **avulsion** | **45** | **2018.4222** |
| **avulsion injury** | **11** | **2017.9091** |
| **axillary nerve** | **17** | **2019.4118** |
| **biceps** | **33** | **2019.5152** |
| **biceps muscle** | **21** | **2019.9048** |
| **brachial plexus** | **59** | **2019.5932** |
| **brachial plexus injury** | **51** | **2020.6275** |
| **brachial-plexus** | **31** | **2020.5161** |
| **brachial-plexus injuries** | **22** | **2019.5** |
| **brachial-plexus injury** | **16** | **2019.125** |
| **branch** | **29** | **2020.1034** |
| **c5** | **18** | **2018.8333** |
| **c7 transfer** | **10** | **2018.1** |
| **children** | **20** | **2018.15** |
| **contralateral c7 transfer** | **15** | **2019.0667** |
| **elbow flexion** | **43** | **2018.7674** |
| **head** | **11** | **2019** |
| **intercostal** | **17** | **2019.8824** |
| **intercostal nerve** | **16** | **2019.6875** |
| **long head** | **22** | **2019.3182** |
| **median nerve** | **51** | **2018.9216** |
| **musculocutaneous nerve** | **41** | **2019.3902** |
| **nerve transfer** | **239** | **2020.2259** |
| **neurotization** | **83** | **2019.494** |
| **part** | **22** | **2019.9545** |
| **phrenic nerve** | **16** | **2018.5** |
| **prespinal route** | **15** | **2019.5333** |
| **restoration** | **80** | **2020.1125** |
| **root avulsion** | **11** | **2018.5455** |
| **root transfer** | **27** | **2019.2593** |
| **shoulder** | **36** | **2020.1111** |
| **spinal accessory nerve** | **32** | **2019.6562** |
| **stroke** | **10** | **2020.6** |
| **suprascapular nerve** | **32** | **2020.1562** |
| **triceps** | **31** | **2020.0323** |
| **upper extremity** | **24** | **2018.375** |
| **anastomosis** | **33** | **2019.9697** |
| **anatomy** | **33** | **2019.9091** |
| **donor** | **11** | **2020.7273** |
| **experience** | **17** | **2018.9412** |
| **face** | **10** | **2019.8** |
| **facial nerve** | **19** | **2019.1053** |
| **facial palsy** | **14** | **2020.6429** |
| **facial paralysis** | **28** | **2019.2857** |
| **facial reanimation** | **15** | **2020** |
| **facial-nerve** | **13** | **2020** |
| **hypoglossal nerve** | **11** | **2018.6364** |
| **innervation** | **15** | **2019.1333** |
| **masseteric nerve** | **17** | **2019.4706** |
| **motor-nerve** | **22** | **2019.6364** |
| **muscle** | **68** | **2020.2059** |
| **muscle transfer** | **15** | **2019.4667** |
| **neurorrhaphy** | **20** | **2020** |
| **palsy** | **74** | **2019.6081** |
| **paralysis** | **42** | **2020.5952** |
| **quality-of-life** | **15** | **2020.6** |
| **reanimation** | **47** | **2020.2979** |
| **rehabilitation** | **29** | **2019.2069** |
| **smile** | **20** | **2020.15** |
| **surgery** | **55** | **2019.5636** |
| **anterior interosseous nerve** | **31** | **2020.5484** |
| **anterior transposition** | **12** | **2019.4167** |
| **cubital tunnel syndrome** | **13** | **2018.4615** |
| **cubital tunnel-syndrome** | **12** | **2018.3333** |
| **decompression** | **10** | **2020.1** |
| **elbow** | **37** | **2019.1351** |
| **femoral nerve** | **10** | **2020.1** |
| **foot** | **11** | **2017.8182** |
| **foot drop** | **11** | **2019.8182** |
| **injuries** | **57** | **2019.9123** |
| **lesions** | **37** | **2019.3784** |
| **management** | **57** | **2019.807** |
| **motor branch** | **30** | **2020.2333** |
| **nerve** | **17** | **2020.5882** |
| **neurolysis** | **10** | **2020.7** |
| **neuropathy** | **24** | **2019.3333** |
| **outcomes** | **100** | **2019.97** |
| **pain** | **12** | **2019.75** |
| **resection** | **10** | **2018.5** |
| **simple decompression** | **13** | **2021.3846** |
| **surgical-treatment** | **23** | **2018.913** |
| **tibial nerve** | **18** | **2020.2778** |
| **transposition** | **21** | **2018.9524** |
| **ulnar nerve** | **91** | **2019.6264** |
| **branches** | **12** | **2020.5833** |
| **elbow extension** | **14** | **2018.5714** |
| **electromyography** | **11** | **2019.4545** |
| **finger extension** | **19** | **2020** |
| **flexion** | **16** | **2019.6875** |
| **hand** | **19** | **2019.5263** |
| **hand function** | **18** | **2019.5** |
| **nerve transfer surgery** | **10** | **2020.8** |
| **nerve transfers** | **15** | **2020.9333** |
| **posterior interosseous nerve** | **10** | **2021.2** |
| **radial nerve** | **15** | **2019.0667** |
| **recovery** | **73** | **2020.5479** |
| **reliability** | **10** | **2019.2** |
|  |  |  |
| **spinal cord injury** | **33** | **2017.8788** |
| **spinal-cord-injury** | **19** | **2019.8947** |
| **tendon transfer** | **10** | **2020.6** |
| **tetraplegia** | **27** | **2020.5185** |
| **upper-limb** | **12** | **2020.9167** |
